# Use and Perceptions of Telehealth by Pediatric Occupational Therapists Post COVID-19 Pandemic

**DOI:** 10.5195/ijt.2024.6655

**Published:** 2025-01-15

**Authors:** Alissa R. Baker, Erin R. Barents, Anna G. Cole, Alyssa L. Klaver, Kathie Van Kampen, Lucile M. Webb, Kallen A. Wolfer

**Affiliations:** Department of Occupational Therapy, Western Michigan University, Kalamazoo, Michigan, USA

**Keywords:** Occupational therapy, post-COVID-19, Rationale, Survey, Telehealth

## Abstract

Telehealth was widely adopted in pediatric occupational therapy practice during the COVID-19 pandemic; however, there is limited knowledge on telehealth use post-pandemic. This study collected 132 responses to a mixed-methods survey from March-October 2023 to explore pediatric occupational therapists’ current use and perceptions on telehealth. Results indicated that over half of respondents continue to use telehealth. Frequency of use was differentiated by setting, with a significantly lower proportion of school-based respondents using telehealth compared to outpatient (p<.001) or early intervention (p<.001). The median rate of use was low with 10% of services delivered via telehealth. Respondents view telehealth as moderately effective and satisfactory. Rationale for use of telehealth included parent coaching, access to the natural environment, caregiver involvement, travel convenience, and to accommodate family illness. Challenges included lack of child engagement, limited parent involvement, and regulatory barriers.

The use of telehealth to provide services for various populations grew tremendously beginning in early 2020 as quarantines and isolation were implemented due to the COVID-19 pandemic (Garfan et al., 2021). In occupational therapy, telehealth has been utilized across a variety of pediatric populations, including those with autism spectrum disorder (ASD; Angell et al., 2023; Jamali et al., 2022; Little et al., 2023), cerebral palsy (Tanner et al., 2023), feeding difficulties (Hladik et al., 2023; Tanner et al., 2023), attention-deficit/hyperactivity disorder (Pijarnvanit & Sriphetcharawut, 2023), and refugee children (Belhan Çelik et al., 2022). Additionally, telehealth has been implemented by occupational therapists in different practice settings, such as school-based (Langbecker et al., 2019; Murphy et al., 2021), early intervention (Bravo et al., 2022; Kronberg et al., 2021), and outpatient (Bican et al., 2021; Tanner et al., 2023; Tenforde et al., 2020). Various occupational therapy assessments have been administered via telehealth, most commonly the Canadian Occupational Performance Measure (COPM; Law et al., 2019) (Jamali et al., 2022; Tanner et al., 2021).

Telehealth has been shown to be highly satisfying for both caregivers and occupational therapists (Little et al., 2023; Smith et al., 2023; Tkach & Earwood, 2023). Both groups reported that telehealth use improved caregiver collaboration, engagement, and empowerment due to a greater emphasis on caregiver coaching (Angell et al., 2024; Hladik et al., 2023; Smith et al., 2023). Further, telehealth appears to be more accessible for caregivers due to scheduling flexibility and reduced transportation demands (Angell et al., 2023; Bravo et al., 2022; Smith et al., 2023). In addition to these caregiver benefits, telehealth can decrease overall costs associated with therapy services (Angell et al., 2023; Little, Wallisch et al., 2018). While most caregivers reported that telehealth improved their self-efficacy and decreased their stress levels (Tkach & Earwood, 2023; Wallisch et al., 2019), some caregivers have reported increased stress and risk of burnout with use of telehealth due to added responsibilities of the caregiver in managing the occupational therapy session (Angell et al., 2023; Bravo et al., 2022; Tkach & Earwood, 2024). Other commonly reported barriers to telehealth use include difficulty attaining and maintaining child engagement, limited access to equipment, inability to provide hands-on support, and technical difficulties (Angell et al., 2023; Bravo et al., 2022; Hladik et al., 2023).

More information is needed on how telehealth is used post-COVID-19 pandemic, as uncertainty remains on treatment outcomes compared to in-person services (Zhang et al., 2024). Understanding how pediatric occupational therapists use telehealth will provide important insight into their clinical reasoning process to make decisions about service delivery method and patient care (Schell, 2014). As clinical reasoning is strongly influenced by experience, therapists’ use of telehealth may influence their current perception and use of telehealth services (Berg et al., 2021). Exploration of current telehealth use, perceptions, and rationale will help identify important factors and reasoning processes for other therapists to consider when choosing a service delivery method.

This project's purpose was to identify current use and perceptions of telehealth among pediatric occupational therapists post-COVID-19 pandemic. Specifically, this project describes the use of telehealth and therapists’ perceptions and rationale for using a telehealth service delivery model with pediatric clients. This study aimed to answer the following questions: What is the rate and frequency of telehealth use by pediatric occupational therapists following the end of the COVID-19 restrictions? What are therapists’ perceptions of and rationale for using a telehealth service delivery model with pediatric clients?

## Methods

### Design

We used a cross-sectional mixed methods survey design to explore pediatric occupational therapists’ perceptions and frequency of telehealth use. The survey was administered via Qualtrics. Study methods were submitted and approved by Western Michigan University's Institutional Review Board.

### Participants

Participants of this study were recruited via convenience and snowball sampling. We disseminated the survey via email and social media to state occupational therapy associations, occupational therapy social media accounts and groups, personal social media accounts, and personal emails. Inclusion criteria included: current registered occupational therapists through the National Board Certification in Occupational Therapy (NBCOT) with at least 6 months of experience who consider their primary practice area in one of the following pediatric settings; early intervention (IDEA Part C/birth-3 years), school-based (IDEA Part B), outpatient (birth-18 years), and/or home health (birth-18 years). Of the 156 responses, 24 responses were removed due to being incomplete. As a result, 132 responses were included in the analysis.

### Survey

We developed survey questions as a research team with a focus on the following four areas: participant demographics, current use, perceptions, and rationale. After an initial literature review, we compiled a comprehensive list of unanswered or partially answered questions and discussed how these questions related to our original research question. For example, common barriers and supports for telehealth were compiled from multiple articles and combined into a series of Likert items. We discussed, eliminated, and revised the questions as a group to create a concise question set to increase survey completion rate. These questions were pilot tested by three pediatric occupational therapists working in different practice settings, which was then followed by a second set of revisions.

The final survey was comprised of 17 questions: 14 closed and three open responses (see Appendix). Closed response questions included participant demographics (e.g., years of experience, practice setting), rate of telehealth use, client demographics (e.g., common diagnoses and age), and a series of Likert items related to therapists’ perceived satisfaction, effectiveness, barriers, and facilitators with their telehealth use. The open response questions allowed participants to describe their rationale for telehealth use or disuse, their plans to use telehealth in the future, and any additional comments on their use of telehealth within their pediatric occupational therapy practice. The survey was open from March 16 to October 7, 2023.

### Data Analysis

#### Quantitative Methods

Descriptive analyses including therapist demographics, use of telehealth (i.e., frequency, client ages, and client diagnoses), satisfaction and perceived effectiveness of telehealth, and supports and barriers to telehealth use were conducted using Excel for Mac 16.65 (Microsoft Corporation, Redmond, WA, USA). Inferential analyses were conducted using SPSS 28.0 (IBM Corporation, Armonk, NY, USA). Specifically, telehealth use based on practice settings was analyzed using Fisher's exact test (2xc) due to an expected frequency count of less than five for the home health setting. Perceptions and satisfaction of telehealth were analyzed with respondents grouped by “yes, use telehealth” or “no, do not use telehealth” using the Mann-Whitney U test due to use of ordinal level data. An alpha level of .05 was used for all inferential tests. Responses that completed at least the first two sections (demographics and telehealth use) of the survey were included in the analysis.

#### Qualitative Methods

Qualitative data from the open response questions were coded and grouped into themes via inductive content analysis (Vears & Gillam, 2022). A team of three read all open-ended responses independently and created themes based on commonalities between responses. The team then compared and finalized themes with each other and independently sorted each response into one or multiple thematic categories. Two team members then cross-checked each coding for agreement. In the case of a discrepancy in coding, the third team member made the final decision.

## Results

### Demographics

There were 156 responses to the survey, 132 of which were quality responses. Among the occupational therapists, 17% had received a bachelor's degree, 63% master's degree, 18% doctor of occupational therapy degree, and 2% PhD. Participants’ locations by state were grouped following the US Census Regions (US CensU.S. Bureau, 2021). Most participants were from the Midwest (48%), followed by the South (18%), the West (18%), and the Northeast (16%). Participants also reported the size of their current location, with 34% practicing in large towns or suburbs (20,000-99,999 people), 21% large cities (300,000-999,999 people), cities (100,000-299,999), 14% metropolitan areas (1 million or more people), and 13% villages or towns (less than 20,000 people). Regarding practice setting, 41% of occupational therapists stated that they worked in an outpatient setting (including private practice and hospital based), 30% in school-based, 21% in early intervention, 5% in home health. The remaining 3% of participants were working in other practice settings including academia. Most participants (72%) reported working full-time positions, 18% part-time, 6% pro re nata (PRN, i.e., as needed), and 4% of participants reported their employment status as ‘other’.

### Description of Telehealth Use

**Figure 1.**
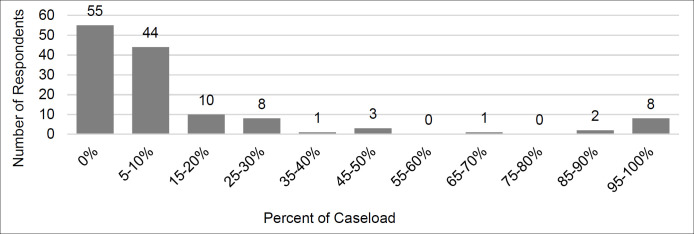
Rates of Telehealth Use

Figure 1 describes the rates of telehealth use. Of the 132 occupational therapists surveyed, 58% (n= 77) stated that they were using telehealth in their practice. Of these 77 occupational therapists that use telehealth, the median amount of usage within their practice was 10%. When asked about the diagnoses and/or conditions of clients receiving telehealth, most therapists reported using telehealth with clients with developmental delays (65%), ASD (64%), and sensory processing disorders (56%). Additionally, 45% of therapists indicated they use telehealth for children with mental health disorders, 35% with feeding difficulties, 24% with cerebral palsy, 16% with congenital conditions, and 11% with orthopedic conditions.

### Practice Setting and Telehealth Use

Fisher's exact test (2xc) was conducted between practice settings and telehealth use, with the exclusion of respondents working in academia or semi-retired due to low number of responses (Table 1). The relationship between practice setting and telehealth use was significant, *χ*^2^ (3, *N*=128) = 21.252, *p*<.001. Post hoc analysis included multiple Fisher's exact tests (2×2) with a Bonferroni correction. The proportion of respondents using telehealth was significantly lower in schools compared to either outpatient settings (*p*<.001) or early intervention (*p*<.001). The proportion of respondents using telehealth was not significantly different between community based, early intervention, and home health (*p*>.05). There was no significant difference between the proportion of respondents using telehealth in home health and schools (*p*>.05).

**Table 1:** Frequencies for Practice Setting and Telehealth Use

	Outpatient	Early Intervention	Home Health	School
	*n* (%)	*n* (%)	*n* (%)	*n* (%)
Use telehealth	39 (72.2)	20 (71.4)	3 (42.9)	11 (28.2)
Do not use telehealth	15 (27.8)	8 (28.6)	4 (57.1)	28 (71.8)

### Satisfaction and Perceived Effectiveness of Telehealth

Examination of perceived effectiveness indicated that 17 (14%) participants found it very effective, 49 (40%) moderately effective, 53 (43%) slightly effective, and 3 (2%) not effective. When asked about satisfaction with telehealth, 16 (13%) participants were extremely satisfied, 47 (39%) somewhat satisfied, 21 (17%) neither satisfied or dissatisfied, 33 (11%) somewhat dissatisfied, and 5 (4%) extremely dissatisfied. A Mann-Whitney U test was conducted to determine if there was a difference between the perception of individuals who use telehealth versus those who do not. There was statistically significant evidence that there was not an equal distribution of perception of effectiveness or satisfaction across the use of telehealth. Specifically, individuals who used telehealth had higher perceived satisfaction (*Mdn*=4) than those who did not use telehealth (*Mdn*=2.5), *U* = 965.5, *z* = 4.54, *p*<0.001. Individuals who used telehealth responded with a higher perceived effectiveness of telehealth (*Mdn*=3), than those who did not use telehealth (*Mdn*=2), *U* = 990, *z* = 4.57, *p*<0.001.

### Supports for Telehealth Use

Quantitative results indicated that over half of respondents perceived the natural environment (62%), caregiver involvement (60%), and reduced travel (58%) as moderate to strong supports for telehealth use. Personal experience with telehealth was rated as a moderate to strong support by 49% of respondents. Only 39% of respondents indicated that administrative oversight provided moderate to strong support for their telehealth use. The most common qualitative reasons with example quotes provided by occupational therapists for using telehealth are summarized in Table 2. Telehealth was often used when in-person services were not accessible due to health and travel reasons. The ability to access and observe the child in their natural environment was another reported rationale for telehealth use. Telehealth also was a preferred service delivery method when caregivers were directly engaged in the sessions, allowing parent coaching and training to be a major part of the treatment.

**Table 2:** Occupational Therapists’ Rationale for Using Telehealth (n=105)

Category	n	Examples
Health	21	“We often use it if the patient or a family member is sick or COVID exposed to support continuation of services”
		“It is ideal for medically fragile kids.”
		“As a temporary option when a client has a sick family member.”
Travel	19	“We utilize telehealth for families that would have to travel hours to our clinic.”
		“I see patients from all over the state without needing to travel large distances.”
		“I work in a more rural area of the state… some places are mor than a 2-hour drive from me. Families have quick access to evaluations and can start services quicker.”
Natural Environment	13	“I love that I am able to work not only with the child but also with their care givers, in their natural environment, with the material that are authentic to their lives.”
		“Sometimes, treating and seeing kids in their home environment [through telehealth] helps us work through challenges more easily.”
		“Allow for continuity of care to the home environment.”
Caregiver Involvement	11	“Parents are more engaged and learn to embed treatment strategies into daily routines.”
Parent Coaching and Training	11	“I feel most confident using telehealth for parent training and coaching.”
		“Telehealth is an excellent way to provide services for early intervention, particularly for parent coaching. Results have been excellent!”

*Note*. ^*^A 10-response cut-off was used to include the highest frequency and most relevant themes.

### Barriers for Using Telehealth

Quantitative results indicated that the top three factors rated as a strong to moderate barrier included client behavior (64%), caregiver preference (49%), and access to technology 32%). Less than 20% of respondents considered lack of experience (17%), administrative support (14%), and insurance/reimbursement (9%) as significant barriers to telehealth use. Table 3 summarizes the qualitative rationale provided by respondents for not using telehealth. The three most common reasons included difficulty with maintaining child engagement, limited caregiver involvement, and administrative regulations.

**Table 3:** Occupational Therapists’ Rationale for Not Using Telehealth (n=105)

Category	n	Examples
Child Engagement	13	“It creates a barrier between building true rapport and engagement with the child.”
		“The population I am working with has great difficulty engaging over a screen for the length of a full session.”
		“I believe play is the most important aspect of pediatric OT, and that organic play via a screen is very difficult.”
Caregiver Involvement	11	“I've also had clients who were a very poor fit for telehealth, often related to caregivers not having the needed skills or willingness to support the session.”
		“Lack of parent involvement - they are home and busy with other tasks.”
Administrative Regulations	11	“Our early intervention program discontinued all use of telehealth as a method of intervention once COVID restrictions were lifted.”
		“It is no longer allowed in my school system.”

*Note*. ^*^A 10-response cut-off was used to include the highest frequency and most relevant themes.

### Plans for Future Telehealth Use

Of the 101 participants who commented on their plans to use telehealth, 78 comments indicated respondents plan to use telehealth in the future, 49 comments referenced that they plan to use it as a backup option when in-person sessions are not possible, and 15 were not planning to use it at all (Table 4). Plans for future use were varied with an emphasis on finding it beneficial in specific situations such as for caregiver education, homebound clients, treatment in the natural environment, and access for rural clients. Similar themes were identified in comments related to using telehealth as a back-up, including to accommodate for illness or inclement weather in lieu of an in-person session.

**Table 4:** Occupational Therapists’ Future Plans for Using or Not Using Telehealth (n=101)

Future Plans	n	Examples
Do Plan to Use	78	“I will use telehealth for education, service delivery, consultation, collaboration, and assessments as needed”
		“I plan to use it with students who are homebound or per parents request.”
		“I plan to use telehealth to continue feeding services in the natural environment.”
		“We continue to provide telehealth services to children who are not within a reasonable driving distance.”
Only as a Backup	49	“If a child or family member is sick… this could be an option that is more preferable than missing a session.”
		“We use it often when we have snow days or other weather-related issues that impact travel.”
		“Definitely as a backup when there are challenges with doing face to face.”
Do Not Plan to Use	15	“I do not plan on using telehealth services unless a family requests it.”
		“I didn't feel it was effective during COVID closures, and my county doesn't allow it any longer.”
		“I will never use it again.”

*Note*. ^*^A 10-response cut-off was used to include the highest frequency and most relevant themes.

## Discussion

The results of this research study found that most survey respondents (58%) are still using telehealth; however, further analysis indicated that there are significant differences in use across practice settings. A lower proportion of school-based therapists (28%) reported using telehealth compared to outpatient (72%) and early intervention therapists (71%). This is likely explained by administrative regulations that prohibit or restrict the use of telehealth in schools once almost all schools returned to full-time in-person instruction in fall 2021 (U.S. Department of Education, 2022). This aligns with both our quantitative data on frequency of use and multiple qualitative comments from school-based therapists which indicated that “it is no longer allowed” and that since “students are back in school, it makes the most sense to see them in person.” This appears to be reflected in current research as well given that most studies focus on the use of telehealth in early intervention (e.g., Bravo et al., 2022; Cheung et al., 2023; Kronberg et al., 2021) and outpatient settings (e.g., Angell et al., 2023; Little et al., 2023; Tanner et al., 2023). There is less literature on school-based telehealth therapy, and the literature that does exist focuses on the use of telehealth in rural and underserved areas (e.g., Langbecker et al., 2019).

Our results were consistent with past research indicating that therapists use telehealth to provide services to a broad range of clients including those with developmental delays (Bravo et al., 2022; Kronberg et al., 2021), ASD (Angell et al., 2023; Jamali et al., 2022; Little et al., 2023), feeding difficulties (Hladik et al., 2023), and cerebral palsy (Tanner et al., 2023). We also found that many therapists are using telehealth to treat children with sensory processing disorders and mental health conditions. This adds to current research in this area which has been limited to recommended adaptations for providing sensory integration treatment via telehealth (Schiano et al., 2024) and mental health services provided by other healthcare disciplines (Nicholas et al., 2021).

Previous research has primarily reported positive caregiver perceptions of telehealth including high acceptance and satisfaction (Little et al., 2023; Little, Pope, et al., 2018; Smith et al., 2023; Tkach & Earwood, 2023,). A few studies provide a more nuanced approach and include reports of negative caregiver perceptions such as higher levels of burnout (Angell et al., 2023) and overall low satisfaction (Murphy et al., 2021). Our study adds to this data by reporting on therapist perceptions of telehealth effectiveness and satisfaction. As a group, respondents perceived telehealth as only slightly to moderately effective and only somewhat satisfying; however, further analysis highlighted differences in perceptions based on current use of telehealth. Respondents who used telehealth reported higher levels of satisfaction and perceived effectiveness compared to non-telehealth users. Given that multiple studies support the effectiveness of telehealth (Little et al., 2023; Little, Pope, et al., 2018; Smith et al., 2023; Tkach & Earwood, 2023), one explanation for this group difference is that non-telehealth users may have inherent biases about the effectiveness of its use. Another possible explanation is that therapists reported higher satisfaction with services during the COVID-19 pandemic (e.g., Tanner et al., 2021) because it was their only option. Once these restrictions were removed, therapists returned to in-person services to avoid the challenges and barriers associated with telehealth especially if they commonly provided a hands-on intervention such as sensory integration (Angell et al., 2023).

Common rationales provided by participants for using telehealth were consistent with previously reported benefits including relieving travel burden (Bravo et al., 2022), increasing caregiver engagement (Angell et al., 2024; Tkach & Earwood, 2023), and focusing on parent coaching (Hladik et al, 2023; Little, Pope, et al., 2018). By asking about rationale, our study builds on previous research to suggest that therapists may now be intentionally choosing telehealth over in-person services because of these benefits. An emerging rationale found in this study is the use of telehealth to address health concerns. Increased health and safety have been reported as a benefit of telehealth primarily during the COVID-19 pandemic (Angell et al., 2024; Bravo et al., 2022). Our study adds that therapists are now using telehealth as a backup or supplemental option to in-person services when the client or family member is ill. Another emerging theme includes the ability to treat the client in their natural home environment. Past research has often focused on the challenges associated with the client's home environment during telehealth, such as lack of equipment and frequent distractions (Angell et al., 2023); however, our respondents reported the opposite with 62% indicating that the natural environment was a support for using telehealth. While this may still be a barrier for some clients, some therapists not only saw this as a support but further they intentionally chose telehealth because it allows them to “see the student in their natural home environment and to treat the whole child and family” and “allow for continuity of care.”

The most common rationales for not using telehealth were also consistent with previous literature and included difficulties engaging the child and poor parent involvement (Angell et al., 2023; Bravo et al., 2022). Notably, challenges with technology were not a common complaint or rationale in our study which is a shift from previous findings (Bravo et al., 2022; Hladik et al., 2023; Yang et al., 2021). An emerging rationale for not using telehealth added by this study is administrative regulations restricting telehealth use. While the quantitative data suggests that administrative support only limits telehealth use by a small minority (14%), this lack of support is significant for at least some respondents based on their qualitative comments indicating that administrative regulations precluded them from using telehealth as a service delivery method. It is currently unclear based on our study and previous research to what extent these regulations are connected to specific states, practice settings, or client populations.

Given the overall low rate of use (median of 10% of caseload) and qualitative comments, our respondents appear to be intentional and selective about their use of telehealth. Stated rationales for using telehealth align with core tenets of occupational therapy practice including being client centered (e.g., caregiver preferences and involvement) and taking the context into consideration (e.g., natural environment) (American Occupational Therapy Association, 2020). The results from this study provide additional details and suggestions of factors to consider when choosing to use telehealth. Given that caregiver involvement was reported as rationale both for and against telehealth use, a primary consideration may be caregiver preference. Some caregivers may prefer telehealth because it allows for shared involvement of caregiver dyads (Smith et al., 2023) and increased caregiver efficacy (Little, Pope et al., 2018; Tkach & Earwood, 2023). Other caregivers may prefer in-person services because the added roles of technician and implementer add to their stress and risk of burnout (Bravo et al., 2022; Tkach & Earwood, 2024). Therapists should also consider the client's unique factors such as age, condition, and therapy goals. Many respondents acknowledge the benefits of using telehealth to provide caregiver coaching which is consistent with other research (Cheung et al., 2023; Little, Pope, et al., 2018). This also aligns with best practices for early intervention settings where the coaching model is used with caregivers to teach and empower their use of strategies in daily routines (Adams & Tapia, 2013). Finally, therapists should consider the use of telehealth to increase accessibility of services. Telehealth may be a beneficial service delivery method to increase equity and access to therapy services for clients with health concerns (Bravo et al., 2022), limited family resources (Angell et al., 2023), and/or who live in rural areas (Hladik et al., 2023; Rooks-Ellis et al., 2020). These recommendations are consistent with other reports on clinical decision-making guidelines for use of telehealth (Zhang et al., 2024).

## Limitations and Considerations

The results of this study are limited by sample size and spread, potential sample bias due to sampling method, and difficulty with generalization due to practice setting. Directions for future research could include effectiveness of services delivered via telehealth, caregiver perceptions of telehealth, and reimbursement factors for telehealth. One specific reimbursement factor to explore in the future includes a new group of CPT codes, effective January 1, 2024. These codes provide reimbursement for caregiver training, even when the direct client is not present (Karr, 2023). Because the themes of this study emphasized caregiver involvement in the quality of telehealth services, future research could examine the relationship between telehealth and these codes.

## Conclusion

This study adds depth and perspective to the conversation surrounding telehealth in pediatric practice. While telehealth was used by most survey respondents, the median rate of use remains relatively low at 10%. This may suggest that therapists are intentional in their use of telehealth based on caregiver preferences, client factors, and accessibility. Telehealth is perceived as moderately effective and satisfactory; with current use of telehealth reporting higher perceived effectiveness and satisfaction. Given the results of this study, occupational therapy practitioners should carefully consider all relevant factors when deciding if telehealth is the right service delivery model for each client, including caregiver involvement, client engagement, environmental factors, and accessibility. These considerations should be well documented to justify the provision of virtual occupational therapy services not just for convenience, but to provide the most client-centered and effective treatment possible.

## Appendix

### Qualtrics Survey

#### Demographics

1. How many years of experience do you have working in a pediatric occupational therapy setting?
2. What is your highest level of education
  ○ Bachelor's degree
  ○ Master's degree
  ○ Entry-level OTD
  ○ Post-professional OTD/DrOT
  ○ PhD/DSc/EdD
  ○ Other (please indicate)
3. What is your primary practice setting?
  ○ Community-based (including private practice) outpatient
  ○ Hospital-affiliated outpatient
  ○ Early Intervention (birth-3; funded by IDEA Part C)
  ○ Home health
  ○ Other (please indicate)
4. What is your current employment status?
  ○ Full time
  ○ Part time
  ○ PRN (please indicate approximate # of hours per week)
  ○ Other (please explain)
5. In which state do you work?
6. Which of the following best describes the primary location where you practice?
  ○ Metropolitan (1 million or more)
  ○ Large city (300,000-999,999 people)
  ○ City (100,000-299,999)
  ○ Large town or suburb (20,000-99,999 people)
  ○ Village or town (less than 19,999 people)

#### Telehealth Use

7. Are you currently using, or have you used *telehealth* as a service delivery method in the past 3 months to provide pediatric occupational therapy services?
  ○ Yes → proceed with Questions 8–10
  ○ No → skip to Question 11
8. Approximately what percentage of your pediatric services did you provide via *telehealth* within the past 3 months? (sliding scale with 5% increments)
9. What diagnoses apply to the children and/or adolescents that you provided *telehealth* services to in the past 3 months (check all that apply)
  ○ Autism spectrum disorder
  ○ Emotional impairments ad/or mental health disorders
  ○ Developmental delays and/or delayed milestones (including developmental coordination disorder and fine or gross motor delays)
  ○ Cerebral palsy (including other movement disorders)
  ○ Congenital abnormalities (including syndromes, chromosomal abnormalities, and malformations)
  ○ Feeding difficulties
  ○ Musculoskeletal or orthopedic impairment (including torticollis)
  ○ Non-congenital brain injury (including stroke, cancer, concussion, or other brain injury)
  ○ Prematurity or low birth weight
  ○ Sensory processing disorders
  ○ Spinal cord injury
  ○ Other (please indicate):
10. What ages are the children that you provided *telehealth* services to in the past 3 months? (check all that apply)
  ○ Birth – 2 yr 11 mo
  ○ 3 yr – 5 yr 11 mo
  ○ 6 yr – 9 yr 11 mo
  ○ 10 yr – 13 yr 11 mo
  ○ 14 yr – 17 yr 11 mo
  ○ 18 yr +

#### Telehealth Perceptions

11. To what extent do you consider *telehealth* to be an effective service delivery method when working with pediatric clients?
  ○ Not effective at all
  ○ Slightly effective
  ○ Moderately effective
  ○ Very effective
12. How satisfied are you with *telehealth* as a service delivery method when working with pediatric clients?
  ○ Extremely dissatisfied
  ○ Somewhat dissatisfied
  ○ Neither satisfied nor dissatisfied
  ○ Somewhat satisfied
  ○ Extremely satisfied
13. To what extent have the following barriers decreased your likelihood to provide *telehealth* services following the removal of COVID-19 restrictions?
  **Table:**
  	Not at all	A little	A moderate amount	A great deal
  Personal lack of telehealth experience and/or training	◯	◯	◯	◯
  Lack of knowledge or access to technology by the client or caregiver	◯	◯	◯	◯
  Difficulty managing client behavior and/or engagement in a virtual setting	◯	◯	◯	◯
  Challenges with reimbursement and/or insurance coverage	◯	◯	◯	◯
  Limited administrative/supervisor support	◯	◯	◯	◯
  Client/caregiver availability and/or preferences	◯	◯	◯	◯
14. To what extent have the following supports or benefits increased your likelihood to provide *telehealth* services following the removal of COVID-19 restrictions?
  **Table:**
  	Not at all	A little	A moderate amount	A great deal
  Personal lack of telehealth experience and/or training	◯	◯	◯	◯
  Lack of knowledge or access to technology by the client or caregiver	◯	◯	◯	◯
  Difficulty managing client behavior and/or engagement in a virtual setting	◯	◯	◯	◯
  Challenges with reimbursement and/or insurance coverage	◯	◯	◯	◯
  Limited administrative/supervisor support	◯	◯	◯	◯
  Client/caregiver availability and/or preferences	◯	◯	◯	◯

#### Telehealth Rationale

15. Describe your rationale for your current use (or lack of use) of *telehealth* as a service delivery method for pediatric occupational therapy.
16. Describe how you plan to use *telehealth* services in the future.
17. Is there anything else you would like us to know about the use of *telehealth* within pediatric occupational therapy settings?
